# Supplementary material for: Elevated pre-mRNA 3′ end processing activity in cancer cells renders vulnerability to inhibition of cleavage and polyadenylation
Source: Nat Commun. 2023 Aug 1;14:4480. doi: 10.1038/s41467-023-39793-8 (PMC10394034; doi:10.1038/s41467-023-39793-8)
Supplement: Supplementary file 1 — Supplementary Information [file 41467_2023_39793_MOESM1_ESM.pdf]

SUPPLEMENTARY INFORMATION

Supplementary Tables

Supplementary Table 1. Oligonucleotides used in this study.

Name	Sequence
HNRNPD readthrough region qPCR primer, forward	5'- AAACTTGCTCTTCAGATACGGGA
HNRNPD readthrough region qPCR primer, reverse	5'- GTCCAGGGCTTATACCCAAACA
HNRNPD last intron qPCR primer, forward	5'- TTGTAGCCTGCCCAGAGTGA
HNRNPD last exon qPCR primer, reverse	5'- CTTCAGAGGGACCCAACGTC
IRES cloning primer, forward	5'- GCCACCACCTGTTCCTGTAGCTCG AGCTCAAGCTTCGAATTCTGCAG
IRES cloning primer, reverse	5'- GGTTGTGGCCATATTATCATCGTG
mNeonGreen cloning primer, forward #1	5'- ATGATAATATGGCCACAACCACCGG TATGGTGTCCAAGGGCGAAGAGG
mNeonGreen cloning primer, reverse #1	5'- AGAGTCGCGGCCGCTTTACTTGTAC AGCTCGTCCATGCCCATC
mNeonGreen cloning primer, forward #2	5'- CGTGATGGGCATGGACGAGCTGTATAA GTCGAATTCTGCAGTCGACGGTACC -
RiG-AD cloning primer, forward	5'- CGATGCTAGCATGGCCTCCTCCGA GGAC
RiG-AD cloning primer, reverse	5'- CCGGCTTAATTAATAAGATACATT GATGAGTTTGGAC
FIP1L1 cloning primer, forward	5'- ATAGCCGAATTCATCGGCCGGCGA GGTCGAG
FIP1L1 cloning primer, reverse	5'- AGTCGCGGATCCCTATTCTGCAGGT GTAGCTTCGGTG

Supplementary Table 2. Compounds used in synergy analysis.

Compound name	Selleck Catalog Number
Hydroxycamptothecine	S3898
Mitoxantrone	S2485
BAY 1895344	S8666
Topotecan	S1231
Teniposide	S1787
AZD6738	S7693
Cytarabine hydrochloride	S5582
Floxuridine	S1299

Supplementary Fig. 1

a

Ratio of no. of downregulated genes to no. of upregulated, fold change >= 1.2

JTE-607 Conc.	1 µM	10 µM
**HeLa**	1.32	1.23
**HepG2**	1.39	1.37

Ratio of no. of downregulated genes to no. of upregulated, fold change >= 2.0

JTE-607 Conc.	1 µM	10 µM
**HeLa**	1.62	1.57
**HepG2**	1.97	2.06

b

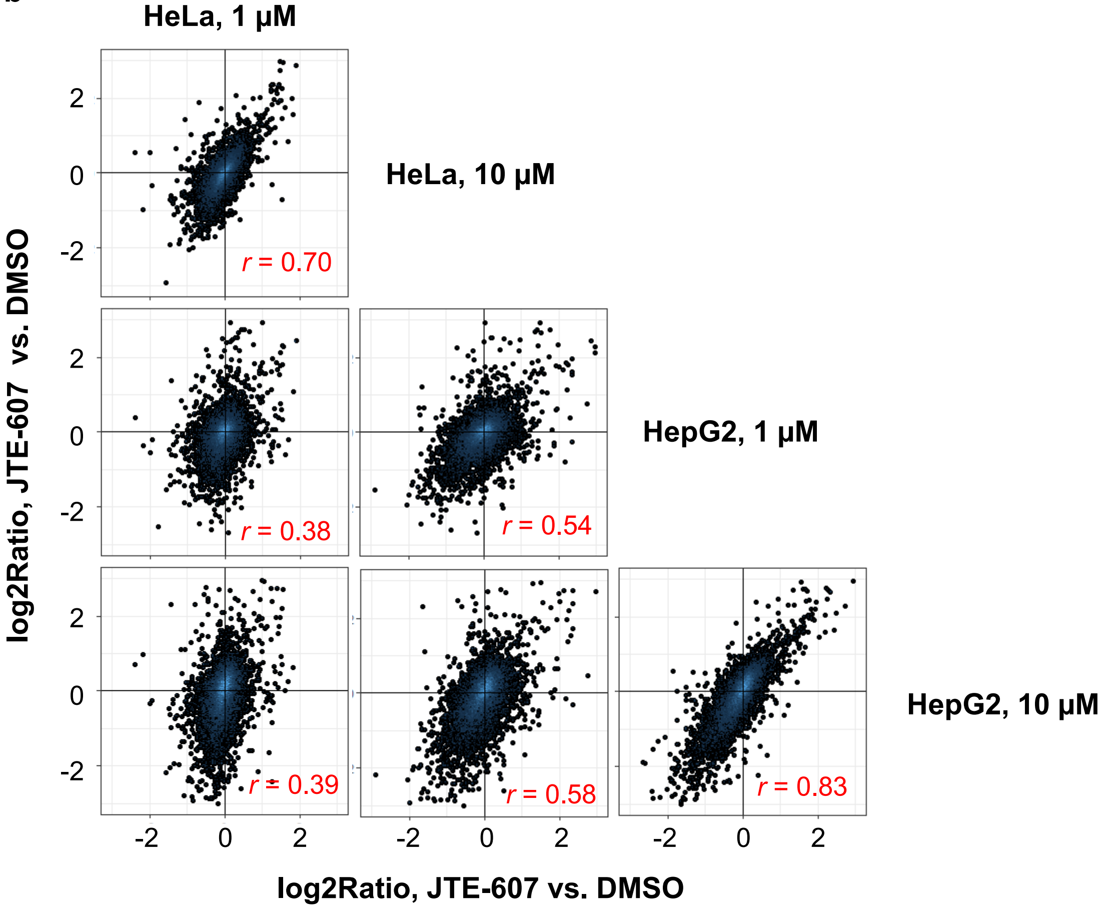

C

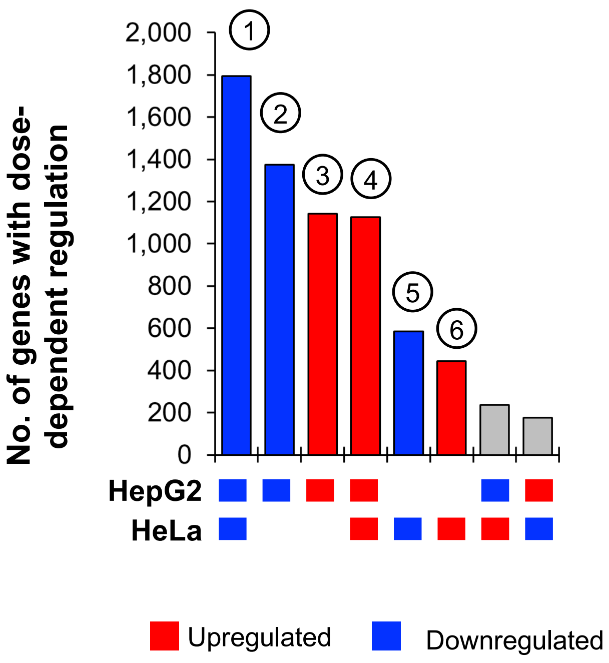

①

GO term	*P*
ncRNA metabolic process	1.45E-07
mitochondrial gene expression	7.41E-06
carbohydrate derivative metabolic process	7.64E-05
carbohydrate metabolic process	1.63E-04
macromolecule methylation	6.00E-04

②

GO term	*P*
heme metabolic process	9.06E-05
response to bacterium	3.76E-04
phosphatidic acid biosynthetic process	4.13E-04
response to cobalamin	1.11E-03
positive regulation of extrinsic apoptotic signaling pathway in absence of ligand	1.45E-03

③

GO term	*P*
negative regulation of execution phase of apoptosis	3.91E-04
mitochondrial ATP synthesis coupled electron transport	4.26E-04
regulation of attachment of spindle microtubules to kinetochore	4.99E-04
attachment of spindle microtubules to kinetochore	6.82E-04
endocrine pancreas development	6.82E-04

④

GO term	*P*
mRNA metabolic process	1.84E-07
negative regulation of macromolecule biosynthetic process	7.74E-06
RNA processing	1.30E-04
positive regulation of transcription by RNA polymerase II	1.49E-04
protein K48-linked ubiquitination	7.39E-04

⑤

GO term	*P*
cellular response to cGMP	3.65E-04
cell proliferation involved in kidney development	4.49E-04
cilium assembly	1.09E-03
tRNA metabolic process	1.23E-03
brain-derived neurotrophic factor receptor signaling pathway	2.08E-03

⑥

GO term	*P*
negative regulation of transcription, DNA-templated	2.19E-04
alcohol metabolic process	2.85E-04
positive regulation of myoblast proliferation	6.21E-04
egg coat formation	1.04E-03
regulation of oxygen metabolic process	1.04E-03

Supplementary Fig. 1. a. Ratio of number of downregulated genes to number of upregulated genes using fold change ≥1.2 (top) or ≥2.0 (bottom). **b.** Scatterplots comparing gene expression changes in HeLa and HepG2 cells treated with 1 µM or 10 µM JTE-607. Pearson correlation coefficient (*r*) is indicated on each plot. A total of 8,585 genes with detectable expression in samples were analyzed. **c.** Gene ontology (GO) analysis result for different groups of genes based on their regulation in HeLa and HepG2 after JTE-607 treatment (Fig. 1e).

Supplementary Fig. 2

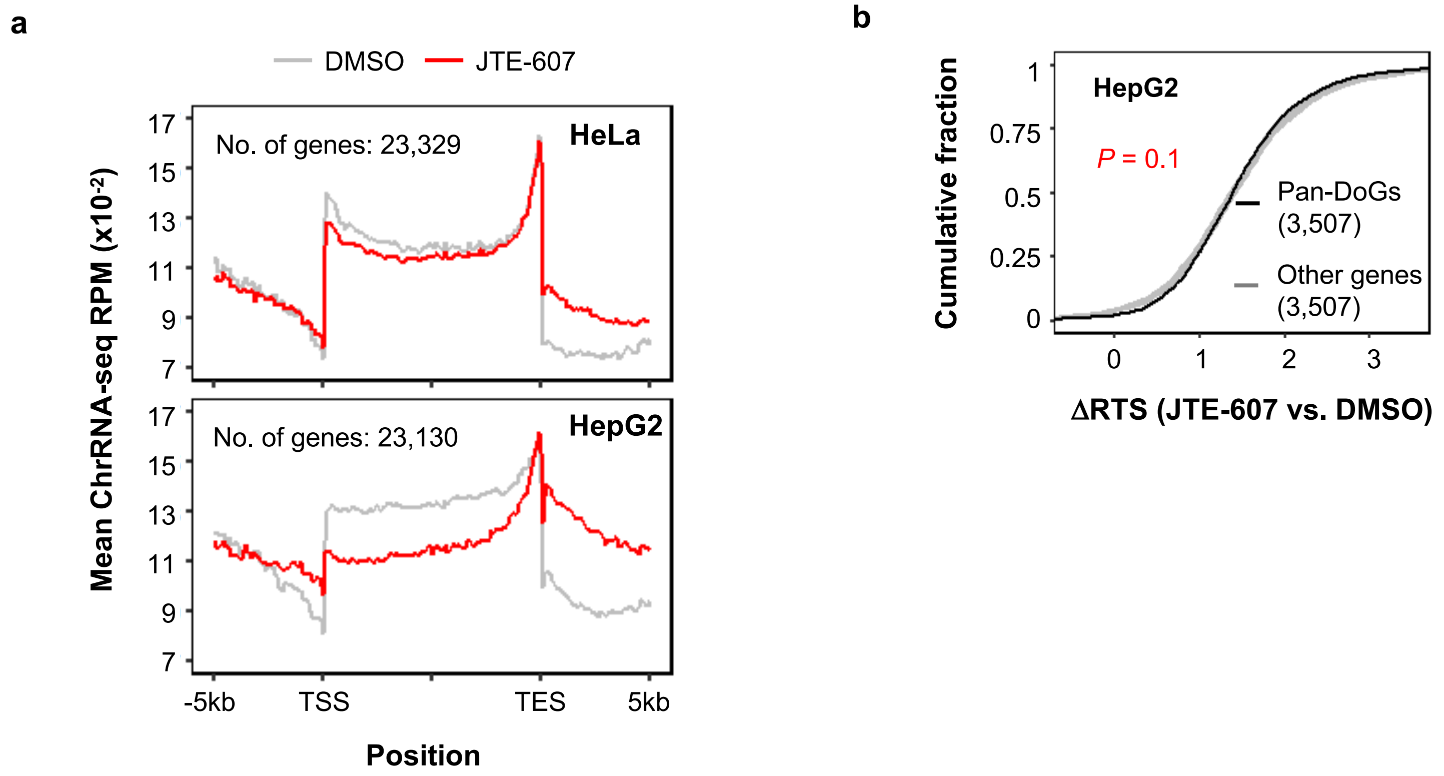

Supplementary Fig. 2. a. Metagene plots of chrRNA-seq reads in genes, including upstream 5 kb, gene body, and downstream 5 kb, in HeLa (top) or HepG2 (bottom) cells after JTE-607 (red line) or DMSO (grey line) treatment. **b.** Comparison of ΔRTS between Pan-DoGs and other genes in JTE-607-treated vs. DMSO-treated HepG2 cells. Other genes are genes randomly selected from non-Pan-DoGs with matched expression (based on percentile). Random selection was carried out 20 times and the median value of the 20 values was used. P-value is based on the K-S test.

Supplementary Fig. 3

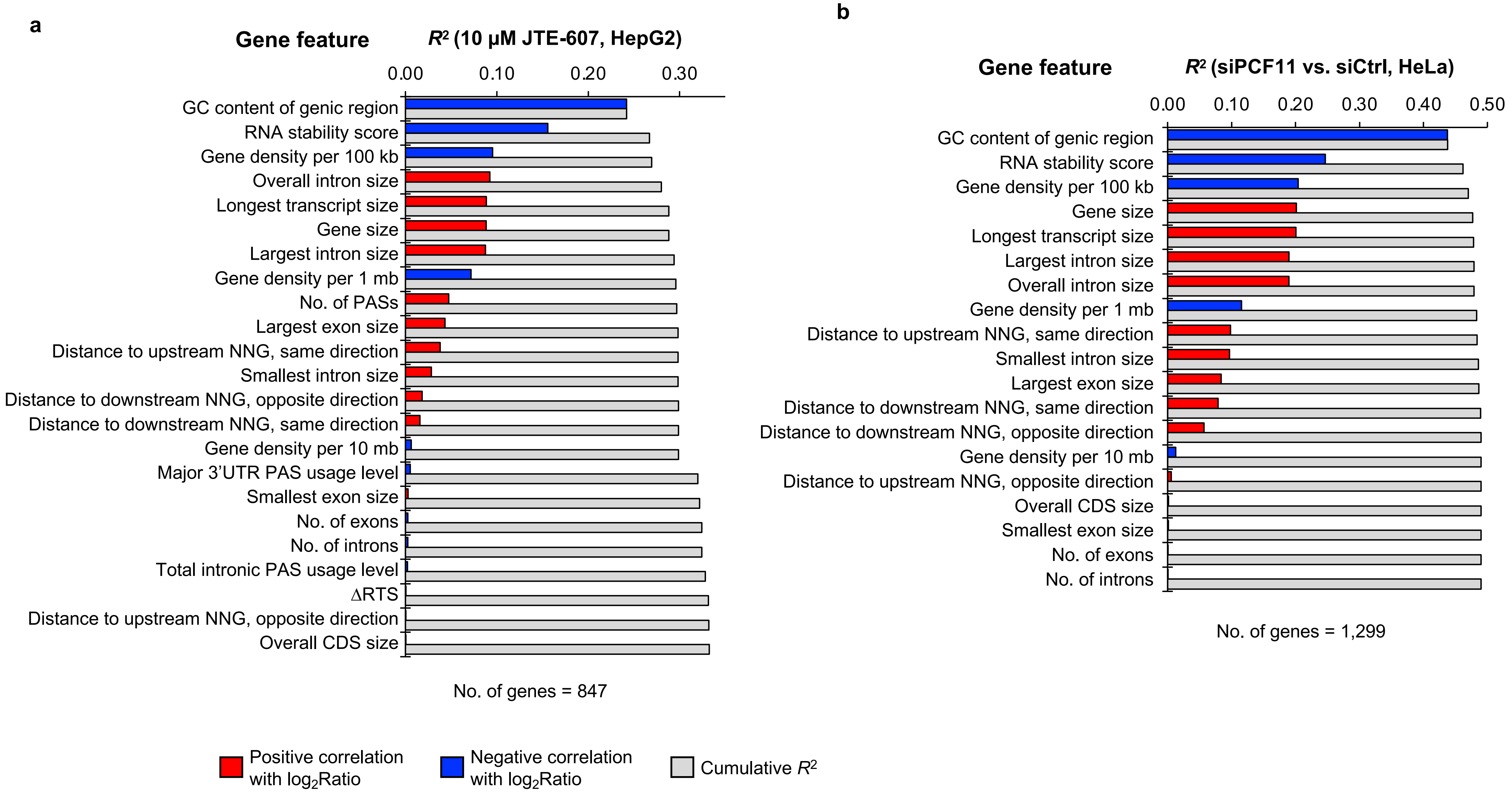

Supplementary Fig. 3. a. All gene features used in this study and their individual and cumulative R^2 values. HepG2 cells with 10 µM JTE-607 treatment is used for analysis. Significantly regulated genes were used. **b.** Gene feature analysis using PCF11 knockdown data (Kamieniarz-Gdula et al.). Significantly regulated genes were used.

Supplementary Fig. 4

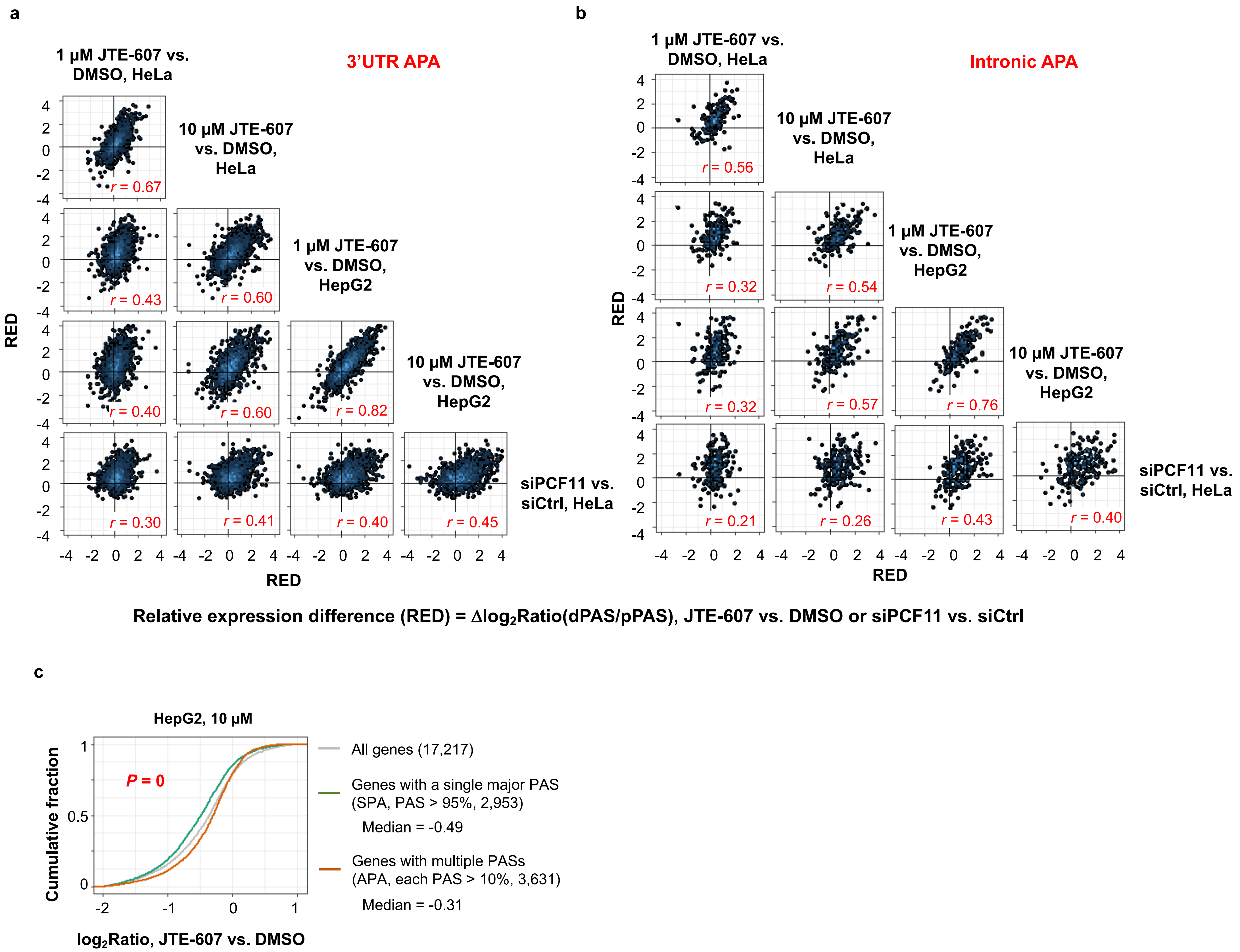

Supplementary Fig. 4. a. Scatterplots comparing 3'UTR APA changes in HeLa and HepG2 cells treated with 1 μM or 10 μM JTE-607, as well as PCF11 siRNA knockdown in HeLa cells (previous study by Kamieniarz-Gdula et al.). A total of 1,198 genes with detectable APA isoform expression in all samples were used. Pearson correlation coefficient (*r*) is indicated on each plot. 3'UTR APA changes are represented by Relative Expression Difference (RED), whose formula is shown. **b.** As in a, except that IPA changes are compared. A total of 207 genes were analyzed. IPA sites are pPAS, and 3'-most exon sites are dPAS. **c.** CDF curves showing gene expression change in HepG2 cells treated with 10 μM JTE-607 for genes with multiple PASs in the last exon (each > 10% of overall isoform expression, APA, red line) and genes with only one major PAS in the last exon (>95% of all isoform expression, SPA, green line). All genes are shown in grey. P-values are based on K-S test. The number of genes for each group is indicated in parathesis.

Supplementary Fig. 5

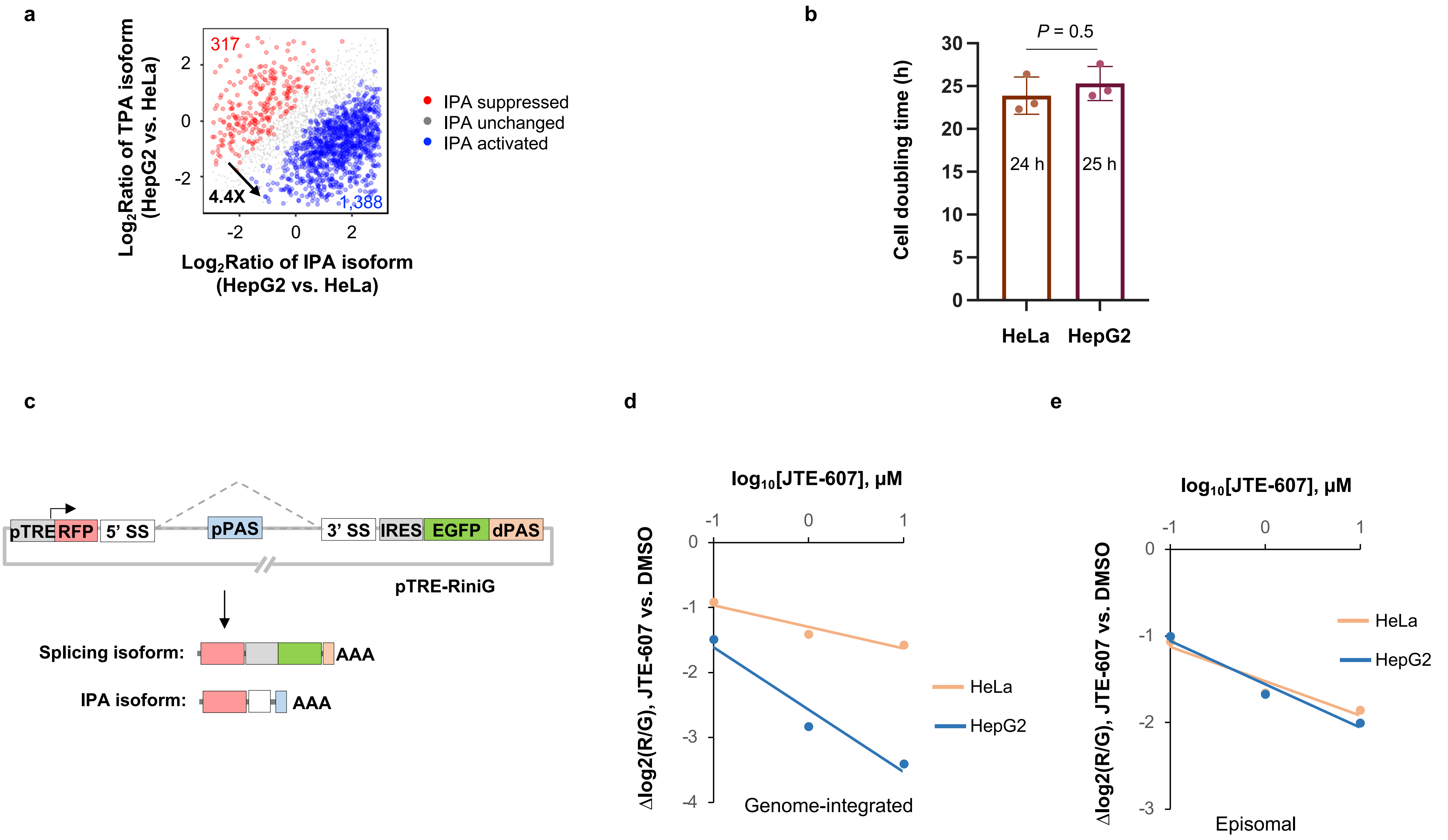

Supplementary Fig. 5. a. Scatter plot comparing IPA and TPA isoform abundance difference between HepG2 cells and HeLa cells. Each dot represents a gene with two selected isoforms. Genes showing IPA suppression and activation in HepG2 cells as compared to HeLa cells are in red and blue, respectively. **b.** Cell doubling time data for HeLa and HepG2 cells. **c.** Schematic of pTRE-RiniG-1600-TT reporter construct to examine IPA regulation. 5' splicing site (5'SS), 3' splice site (3'SS) and pPAS were derived from human CSTF3 gene. Splicing isoform encodes RFP, IRES and EGFP sequences, whereas the IPA isoform encodes RFP and 5'SS. **d.** Suppression of IPA of pTRE-RiniG-1600-TT by JTE-607 in HeLa and HepG2 cells containing genome-integrated construct. **e.** Suppression of IPA of pTRE-RiniG-1600-TT by JTE-607 in HeLa and HepG2 cells transiently transfected with the construct (episomal).

Supplementary Fig. 6

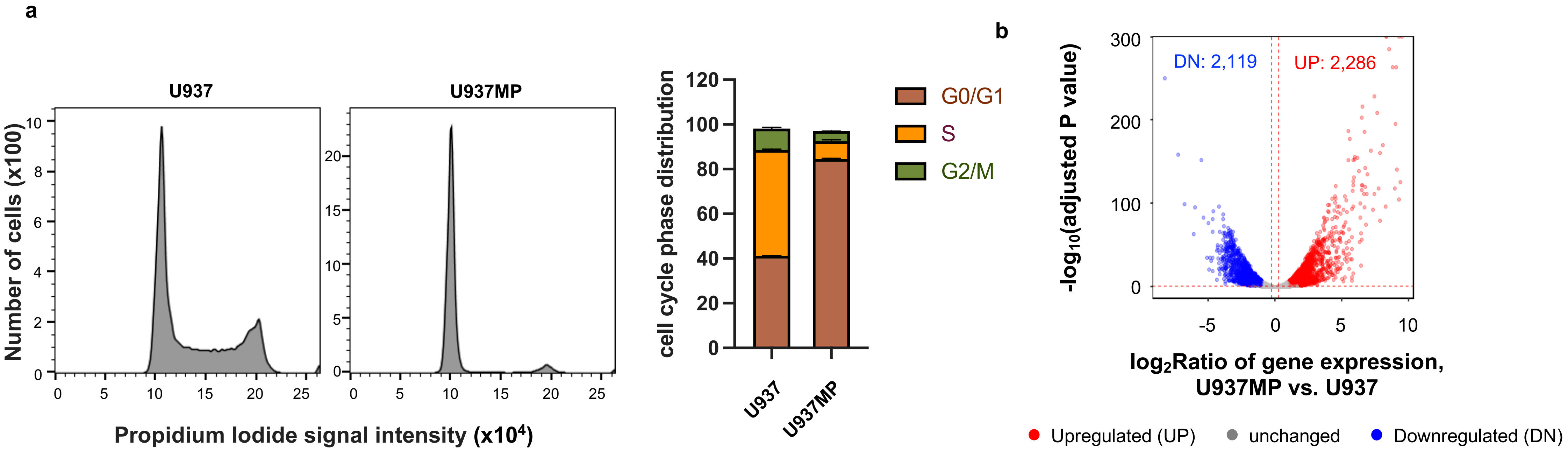

c

GO terms associated with upregulated genes in U937 differentiation

Biological Process	*P*
vesicle-mediated transport	7.95E-46
defense response	4.29E-36
immune response	7.77E-33
cell adhesion	1.27E-27
actin filament-based process	2.44E-25
regulation of immune system process	3.00E-25
locomotion	9.49E-25
regulation of transport	1.91E-24
positive regulation of signaling	1.87E-23
cell activation	1.86E-20
regulation of hydrolase activity	3.72E-19
process utilizing autophagic mechanism	2.16E-18
positive regulation of multicellular organismal process	6.10E-18
secretion by cell	2.53E-17
response to cytokine	1.09E-16
anatomical structure formation involved in morphogenesis	7.90E-16
response to oxygen-containing compound	2.57E-15
negative regulation of multicellular organismal process	3.39E-15
cation transport	4.34E-15
homeostatic process	5.55E-15

GO terms associated with downregulated genes in U937 differentiation

Biological Process	*P*
ribonucleoprotein complex biogenesis	8.91E-61
RNA processing	2.95E-56
chromosome organization	2.47E-45
DNA metabolic process	3.39E-38
cell cycle process	3.16E-30
translation	1.27E-19
RNA localization	1.45E-19
non-membrane-bounded organelle assembly	9.80E-14
methylation	4.50E-10
cellular protein-containing complex assembly	1.32E-08
nucleic acid phosphodiester bond hydrolysis	1.48E-08
nuclear export	7.92E-06
nuclear transport	8.29E-06
nucleobase biosynthetic process	1.24E-05
signal transduction by p53 class mediator	2.68E-05
cellular amino acid metabolic process	3.92E-05
protein-containing complex localization	7.51E-05
DNA-templated transcription, initiation	1.49E-04
nucleoside monophosphate biosynthetic process	1.98E-04
transcription by RNA polymerase I	2.01E-04

Supplementary Fig. 6. a. Cell cycle phase analysis of U937 and U937MP cells. **b.** Differentially expressed genes between U937 and U937MP cells. A total of 13,056 genes were analyzed. **c.** Gene Ontology terms associated with Differentially expressed genes between U937 and U937MPcells.

Supplementary Fig. 7

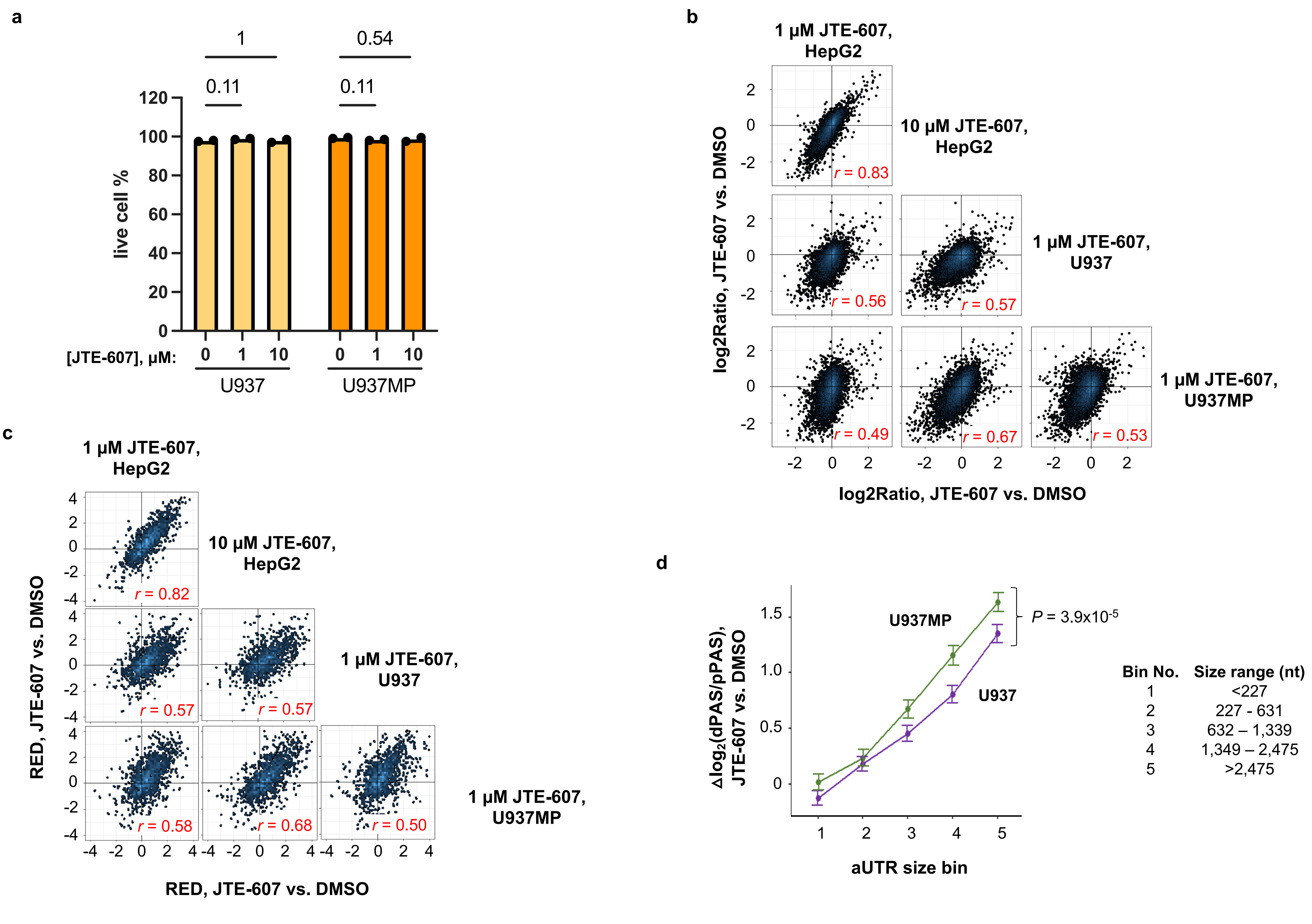

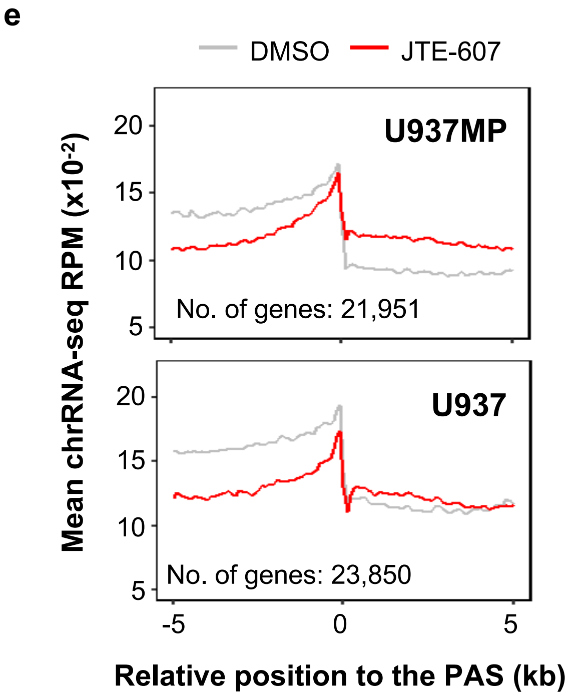

Supplementary Fig. 7. a. Cell survival analysis of U937 and U937MP cells after 7 h of 1 and 10 μM JTE-607 treatment (n = 2). *P* values (t-test) for significance of difference are indicated. **b.** Scatterplots comparing gene expression changes in U937 and U937MP cells treated with 1 μM JTE-607, as well as HepG2 cells treated with 1 μM or 10 μM JTE-607. A total of 8,014 genes with detectable expression in all samples were analyzed. Pearson correlation coefficient (*r*) is indicated on each plot. **c.** As in b, except that 3'UTR APA changes, represented by Relative Expression Difference (RED), are compared. A total of 1,198 genes were analyzed. **d.** 3'UTR lengthening vs. aUTR size in U937MP and U937 cells treated with JTE-607. *P* value (Wilcoxon test) for significance of difference between bin 5s from U937 and U937MP is indicated. A total of 2,205 genes with detectable expression of 3'UTR isoforms were divided into five equally sized bins based on their aUTR size. Error bars are standard error of mean. **e.** Metagene plots of chrRNA-seq reads in the genomic region around the last annotated PAS (+/- 5 kb) in U937MP (top) or U937 (bottom) cells after JTE-607 (red line) or DMSO (grey line) treatment. The chrRNA-seq reads were normalized for each gene. The number of genes used in each plot is indicated.

Supplementary Fig. 8

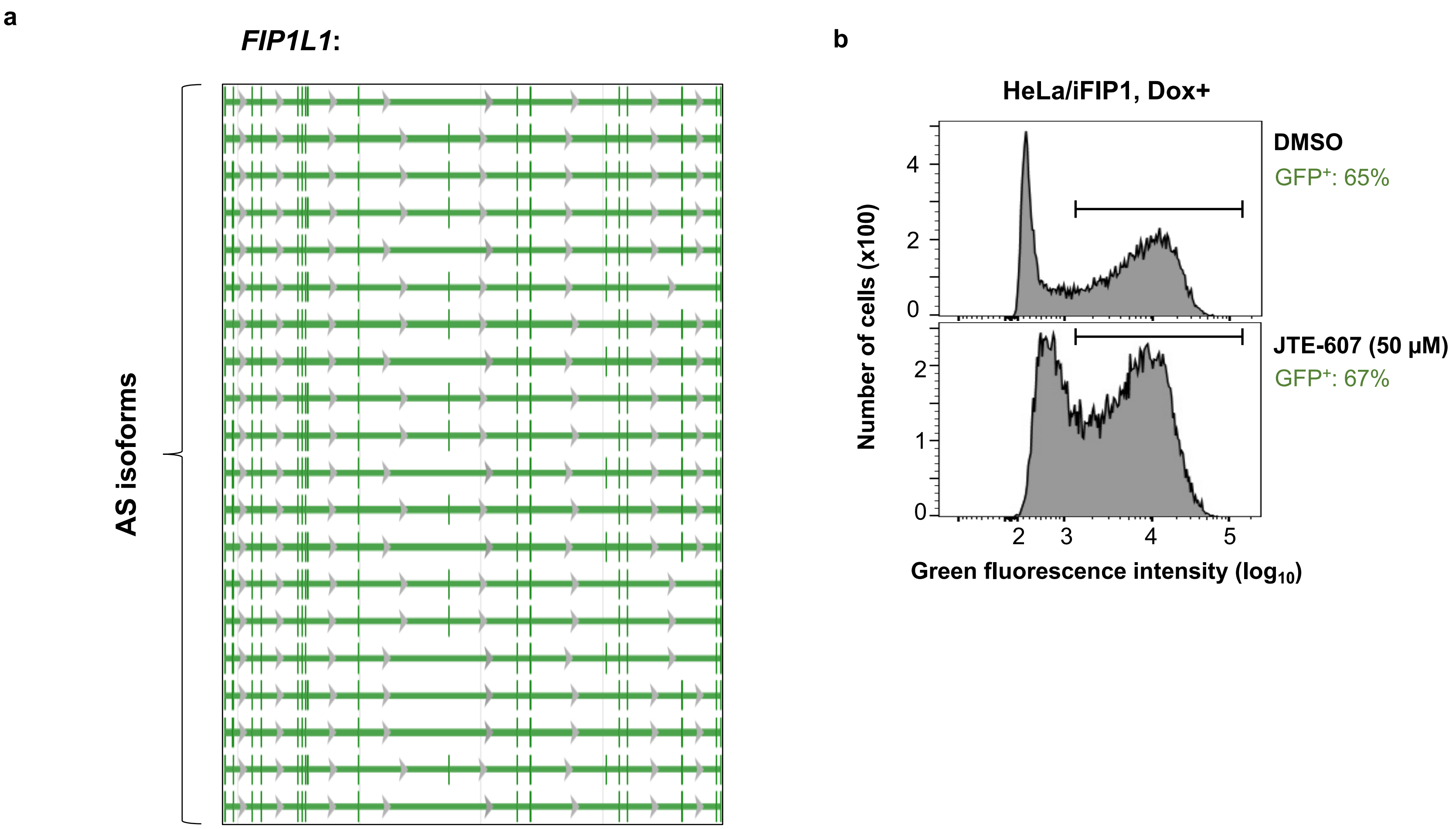

Supplementary Fig. 8. a. Human gene encoding FIP1 (*FIP1L1*) expresses a large number of alternative splicing isoforms as annotated in the NCBI Gene database. **b.** Histograms showing distribution of fluorescence signals of HeLa/iFIP1 cells (Dox+) after DMSO (top) or JTE-607 (bottom) treatment.

Supplementary Fig. 9

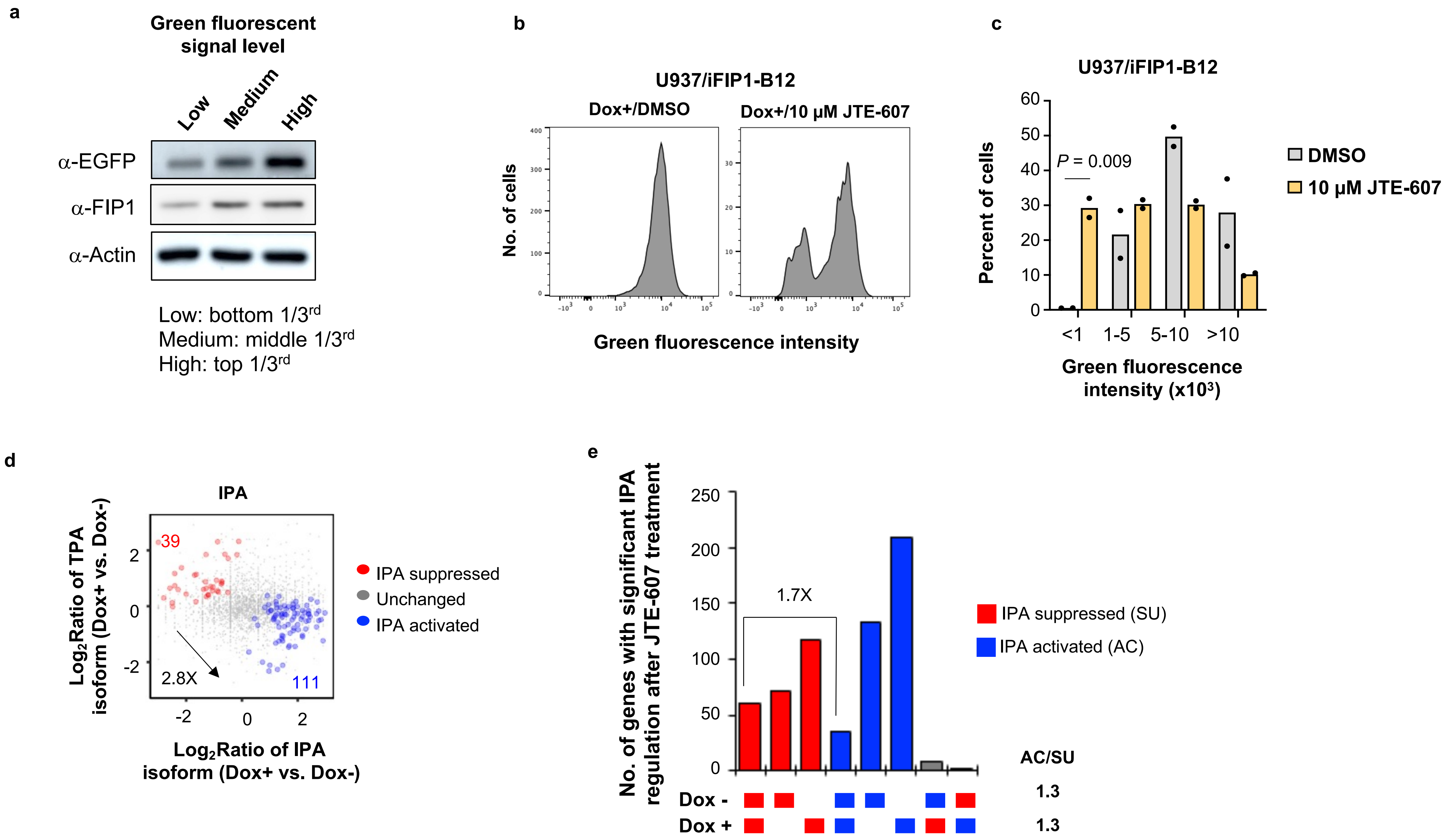

Supplementary Fig. 9. a. Western blot showing FIP1 protein expression levels in U937/iFIP1-A1 cells sorted by green fluorescent signals. **b.** As in Fig. 9c, except that data is based on the U937/iFIP1-B12 clone. **c.** As in Fig. 9d, except that data is based on the U937/iFIP1-B12 clone. **d.** As in Fig. 9g, except that data is based on IPA regulation. **e.** As in Fig. 9j, except that data is based on IPA regulation.

Supplementary Fig. 10

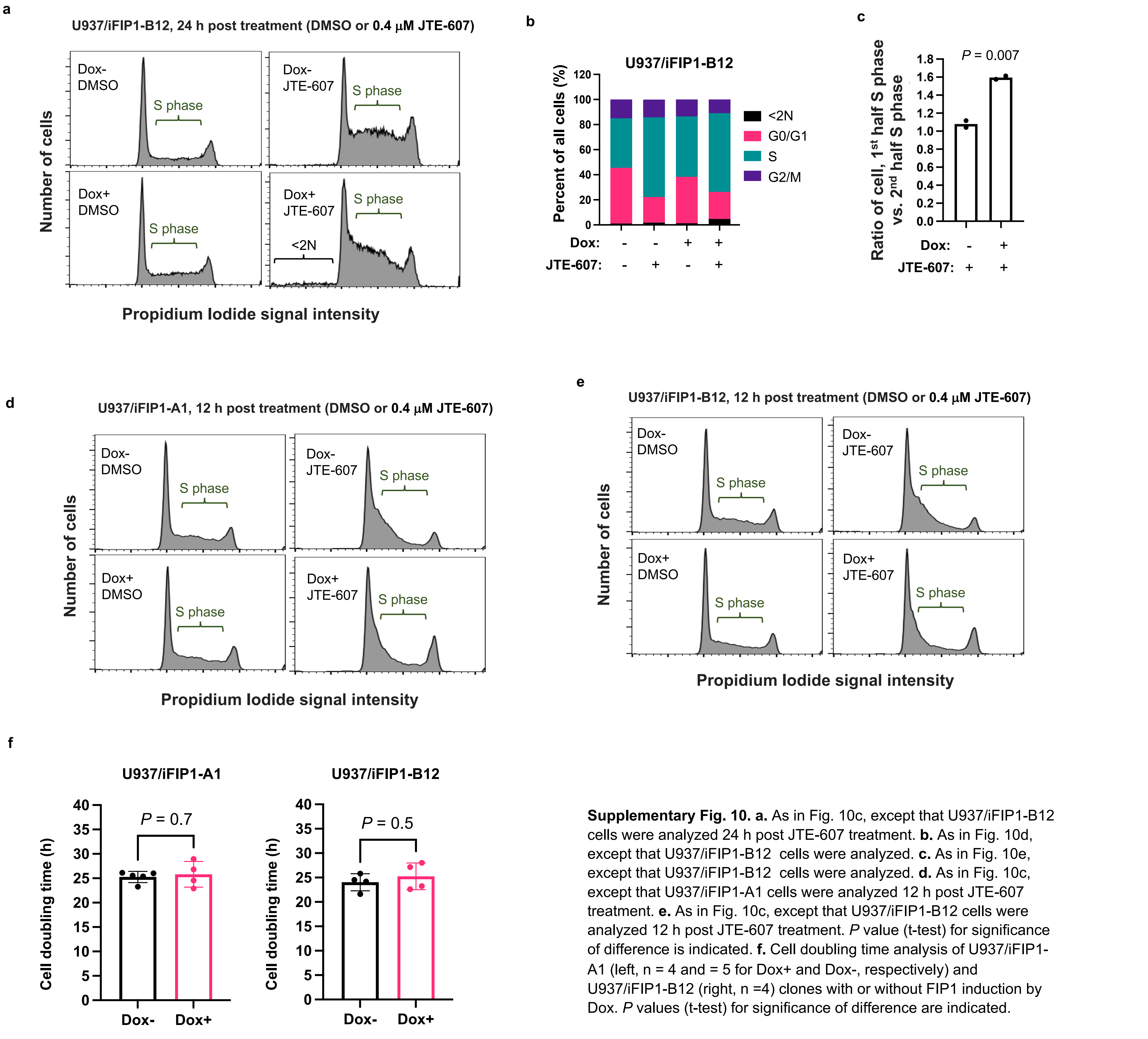

Supplementary Fig. 10. a. As in Fig. 10c, except that U937/iFIP1-B12 cells were analyzed 24 h post JTE-607 treatment. **b.** As in Fig. 10d, except that U937/iFIP1-B12 cells were analyzed. **c.** As in Fig. 10e, except that U937/iFIP1-B12 cells were analyzed. **d.** As in Fig. 10c, except that U937/iFIP1-A1 cells were analyzed 12 h post JTE-607 treatment. **e.** As in Fig. 10c, except that U937/iFIP1-B12 cells were analyzed 12 h post JTE-607 treatment. *P* value (t-test) for significance of difference is indicated. **f.** Cell doubling time analysis of U937/iFIP1-A1 (left, n = 4 and = 5 for Dox+ and Dox-, respectively) and U937/iFIP1-B12 (right, n =4) clones with or without FIP1 induction by Dox. *P* values (t-test) for significance of difference are indicated.

Supplementary Fig. 11

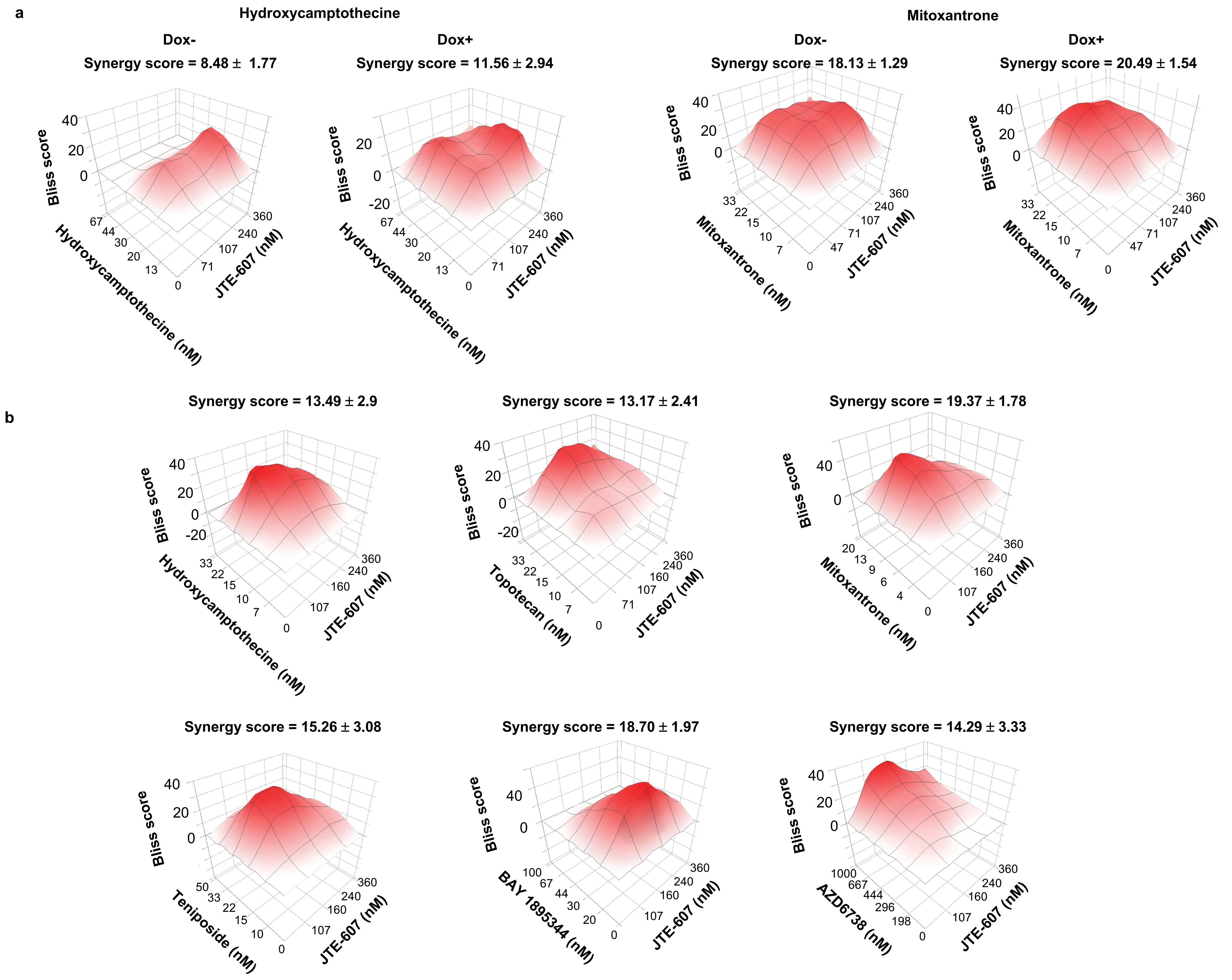

Supplementary Fig. 11. a. Representative synergy plots for hydroxycamptothecin and mitoxantone data shown in Fig. 10f. **b.** Representative synergy plots for data shown in Fig. 10h.

Supplementary Fig. 12

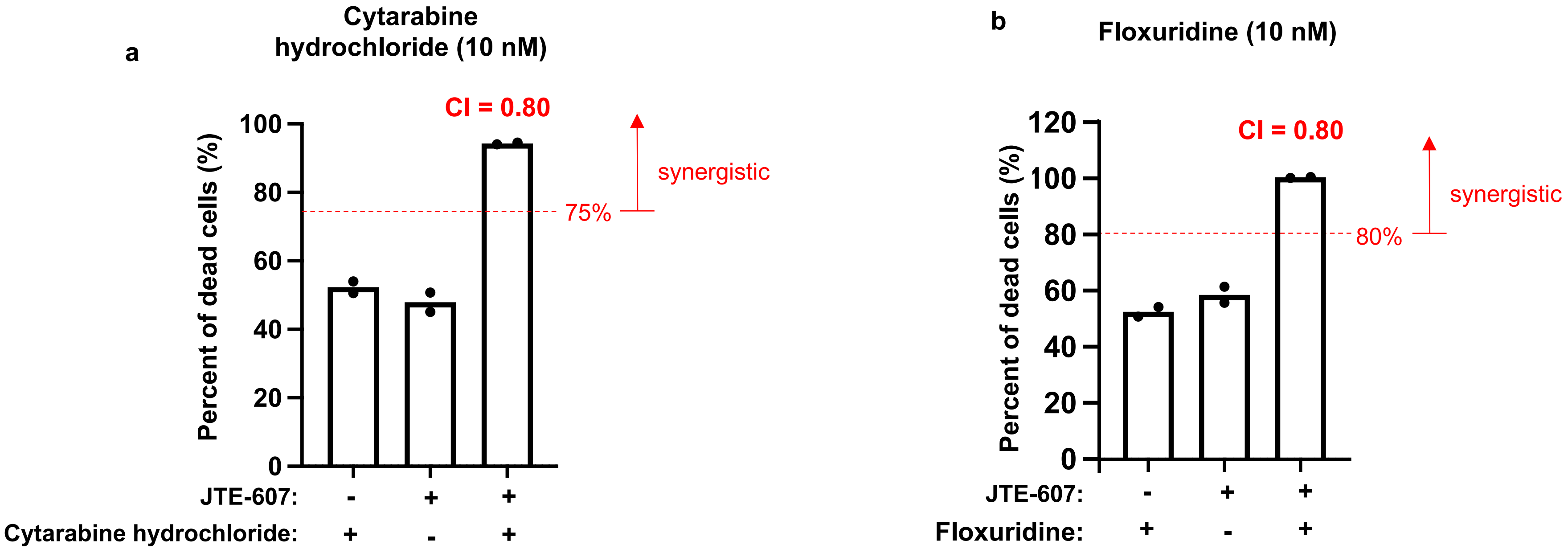

Supplementary Fig. 12. a. Analysis synergy between JTE-607 with cytarabine hydrochloride (n = 2). Synergy was calculated by using the Combination Index (CI) method based on the Bliss Independence model. A CI value < 1 is considered synergistic. The dotting line corresponds to CI value = 1, above which the value is considered synergistic. **b.** As in a., except that data is for floxuridine (n = 2).
